# Patients’ perspective on supposedly patient-relevant process and outcome parameters: a cross-sectional survey within the ‘PRO patients study’

**DOI:** 10.1186/s12913-021-07437-6

**Published:** 2022-01-14

**Authors:** Christine Kersting, Julia Hülsmann, Klaus Weckbecker, Achim Mortsiefer

**Affiliations:** 1 Chair of General Practice II and Patient-Centeredness in Primary Care, Institute of General Practice and Primary Care, Faculty of Health, Witten/Herdecke University, Alfred-Herrhausen-Str. 50, 58448 Witten, Germany; 2 Chair of General Practice I and Interprofessional Care, Institute of General Practice and Primary Care, Faculty of Health, Witten/Herdecke University, Alfred-Herrhausen-Str. 50, 58448 Witten, Germany

**Keywords:** Patient relevance, Patient-relevant outcome, Patient preference, Patient-centered care, Patient involvement

## Abstract

**Background:**

To be able to make informed choices based on their individual preferences, patients need to be adequately informed about treatment options and their potential outcomes. This implies that studies measure the effects of care based on parameters that are relevant to patients. In a previous scoping review, we found a wide variety of supposedly patient-relevant parameters that equally addressed processes and outcomes of care. We were unable to identify a consistent understanding of patient relevance and therefore aimed to develop an empirically based concept including a generic set of patient-relevant parameters. As a first step we evaluated the process and outcome parameters identified in the scoping review from the patients’ perspective.

**Methods:**

We conducted a cross-sectional survey among German general practice patients. Ten research practices of Witten/Herdecke University supported the study. During a two-week period in the fall of 2020, patients willing to participate self-administered a short questionnaire. It evaluated the relevance of the 32 parameters identified in the scoping review on a 5-point Likert scale and offered a free-text field for additional parameters. These free-text answers were inductively categorized by two researchers. Quantitative data were analyzed using descriptive statistics. Bivariate analyses were performed to determine whether there are any correlations between rating a parameter as highly relevant and patients’ characteristics.

**Results:**

Data from 299 patients were eligible for analysis. All outcomes except ‘sexuality’ and ‘frequency of healthcare service utilization’ were rated important. ‘Confidence in therapy’ was rated most important, followed by ‘prevention of comorbidity’ and ‘mobility’. Relevance ratings of five parameters were associated with patients’ age and gender, but not with their chronic status. The free-text analysis revealed 15 additional parameters, 12 of which addressed processes of care, i.e., ‘enough time in physician consultation’.

**Conclusion:**

Patients attach great value to parameters addressing processes of care. It appears as though the way in which patients experience the care process is not less relevant than what comes of it. Relevance ratings were not associated with chronic status, but few parameters were gender- and age-related.

**Trial registration:**

Core Outcome Measures in Effectiveness Trials Initiative, registration number: 1685.

## Background

Patient-centered care implies that patients, their values, preferences, and individual life and health goals are at the heart of care processes and that patients are involved in care decisions [1]. To be able to make a choice based on personal preferences, patients need to be adequately informed about care options and their potential effects, understand the different options, and explore what is most relevant for them [2]. To facilitate this goal, studies must examine the effects of care based on parameters that matter to patients and thereby enable them to make an informed decision.

Recent systematic reviews conclude that outcomes relevant to patients are underrepresented in clinical trials [3–5]. Aiming to explore which parameters are thought to be particularly relevant to patients in current research, we conducted a scoping review on patient relevance that included clinical and epidemiological trials and reviews on such studies from the past 20 years [6]. Interestingly, only one third of the 44 studies analyzed actually applied patient-driven approaches to define and select parameters that are explicitly relevant to patients when designing the study [7–20]. All of these studies were conducted among specific patient groups. Studies addressing generic patient groups and not focusing on one specific disease were underrepresented [4, 5, 21–29]. Overall, the content analysis of the 44 studies yielded neither a consistent terminology or well-founded definition, nor a consistent set of parameters relevant to patients across diseases [6]. Instead, the parameters which were thought to be patient-relevant varied widely from typical clinical trial endpoints like morbidity, mortality, and quality of life, to softer social aspects like, for instance, participation or the ability to work or fulfil social roles. In total, 32 different categories of parameters which addressed processes of care as well as outcomes of care were identified [6]. Another recent review about the use of patient-reported outcomes in core outcome sets found that different outcome sets covered the same domains but recommended different instruments [30]. Even though patient-reported outcomes and patient-relevant outcomes might not necessarily be the same, the findings of both reviews demonstrate inconsistencies in effect measurement which limit the comparability of study results regarding the patient benefit.

In a larger-scale research project called ‘PRO patients study’ we therefore aim to achieve a consensus regarding a concept on patient relevance that is based on the recent literature, considers the patient’s perspective, and is feasible for scientific purposes [31]. This concept will determine the terminology that is most suitable to describe patient-relevant outcomes while also providing insights into the criteria that are appropriate to characterize outcomes relevant to patients in the sense of a definition. Hypothesizing that some parameters are relevant for all patients, regardless of their ailment, the concept will also include a prioritization of parameters that mostly represent generic patient relevance independently of diseases. Based on the results of grading outcomes and on the experiences made during the consensus process, an empirically based methodological framework on how to select and weigh parameters according to patients’ preferences will be derived in the long-term. This framework will be applicable and adaptable to different contexts and will consider not only the process of designing trials, but also that of shared decision-making [31]. Regarding shared decision-making it will help to individually prioritize trial outcomes from the patients’ perspective in order to facilitate the process of exploring what is most relevant for them as also described in other approaches [32, 33].

As a first step of the ‘PRO patients study’ we conducted a cross-sectional survey among German general practice patients to evaluate their views and beliefs concerning different general treatment outcomes that had been considered as relevant to patients in former clinical and epidemiological trials including reviews on such trials [6]. The aim of this survey presented in this paper is to gain insight into how patients evaluate and weigh the different outcome dimensions of medical treatments in order to contribute to an empirically based methodological concept of patient relevance in medical decision-making in the future.

## Methods

This paper is reported in accordance with the STROBE statement [34]. It reports only on the first phase of the ‘PRO patients study’, which consists of four phases in total. Knowing that the outcomes extracted from former studies might not be exhausting, the results of the first phase will provide the basis for a multi-professional group discussion on patient-relevant outcomes in the second step, which will finally lead to a two-round online Delphi consensus process [31]. More details on the method of the whole ‘PRO patients study’ are published in a study protocol [31].

### Study design

The study was designed as a cross-sectional survey.

### Setting and participants

The survey was conducted among German general practice patients. Participants were required to be at least 18 years old and possess sufficient German language skills to answer a written questionnaire. No further inclusion and exclusion criteria were defined, as patients of different age, gender, and diseases were included. In order to obtain meaningful results, the minimum sample size was defined as 100.

Patients were recruited via teaching and research practices of the Chairs of General Practice of Witten/Herdecke University. These practices are affiliated with the University as they support research projects and students’ teaching. They do not, however, differ from other general practices in terms of patient care [35]. All 13 practices that attended the network meeting in the fall of 2020 were asked to support the study and were trained on how to conduct the survey within their practices. For each practice, data were collected during a two-week period between September 28^th^, 2020, and November 13^th^, 2020. During the data collection period, every fifth patient who visited their doctor for a scheduled appointment and met the inclusion criteria was asked to complete the survey. This specific procedure for selecting patients was chosen to prevent convenience sampling of patients by the practice teams. Furthermore, this approach aimed to make the recruitment process and the study conduct feasible for the practices as the questionnaires were completed in the practices’ waiting rooms whose capacities were limited due to the COVID-19 pandemic.

### Variables and data sources

Patients willing to participate self-administered a three-page, anonymous questionnaire. The questionnaire was developed by the authors on the basis of the results from the previous scoping review [6]: All 32 patient-relevant parameters extracted from the clinical trials, epidemiological studies, and reviews analyzed in the scoping review were included in the questionnaire. In order to structure the questionnaire, the parameters were categorized into parameters related to *body and mind* (11 parameters), *personality and social life* (12 parameter), and *diagnosis and care processes* (9 parameters). Patients were asked to rate the overall relevance of each parameter from their individual perspective on a 5-point Likert scale ranging from ‘not relevant’ to ‘highly relevant’. Additionally, a free-text field provided the opportunity to add further relevant parameters. In the first pretest the reliability and validity of items and response categories were evaluated and discussed by the chair’s study group and further scientists of Witten/Herdecke University conducting research in the outpatient setting. After revision, the questionnaire was pretested with the general practitioners and health care assistants who attended the network’s 2020 fall meeting. This approach was considered sufficient as both groups are occupationally experienced in using questions and wordings that are comprehensible for patients.

For patients unwilling to participate, age, gender, chronic disease (yes/no), and occupational status were documented.

### Bias

To minimize the risk of selection bias due to non-response on an item level, the questionnaire provided the possibility to select a neutral answer (*outcome neither relevant nor irrelevant*) and to declare that the responder did not know what a specific parameter meant (*unsure what this parameter means*). Additionally, characteristics of patients unwilling to participate were documented to control for a potential selection bias due to study non-response.

### Statistical methods

Responders and non-responders were compared regarding age, gender, employment, and chronical illness using a t-test for independent samples for continuous variables and a Pearson’s chi-squared test for categorical variables (Fisher’s exact test if cells were < 5) [36]. The nominal significance level for these bivariate analyses was defined as p < 0.05.

The relevance of parameters was determined by a simple frequency calculation and a calculation of mean values for each of the 32 parameters included in the questionnaire. Only valid scorings were included for the analysis of mean values, i.e., answers stating ‘unsure what this parameter means’ were excluded. Parameters that scored ≥ 4 on average were considered relevant. In order to determine whether there were any differences in rating a parameter as highly relevant (yes/no) with regard to age (continuously), gender (male/female), and chronic status (chronically ill/not chronically ill), bivariate analyses were performed using a Mann–Whitney U test for continuous variables and a Pearson’s chi-squared test for categorical variables (Fisher’s exact test if cells were < 5) [36]. In order to control for multiple testing, p-values were corrected by applying the Bonferroni-Holm method [37, 38]. Aiming to additionally identify potential redundancies between parameters and to extract the most important factors, one principal component analysis (PCA) each was performed for parameters related to *body and mind*, *personality and social life*, and *diagnosis and care processes*. In case of Kaiser–Meyer–Olkin measure of sampling adequacy > 0.5 and a significant value for the Bartlett’s sphericity test (< 0.05) correlations between the variables selected were considered sufficient for conducting a PCA [39]. Factors were extracted based on the Kaiser’s criteria (eigenvalue ≥ 1) and the scree-plot [39].

The free-text answers were inductively categorized by two researchers (CK, JH). Simple frequency calculations were then applied.

All statistical analyses were performed using IBM SPSS Statistics for Windows, Version 26 (Armonk, New York: IBM Corp.). Percentages and mean values are reported for valid cases.

## Results

### Participants

**Fig. 1:**
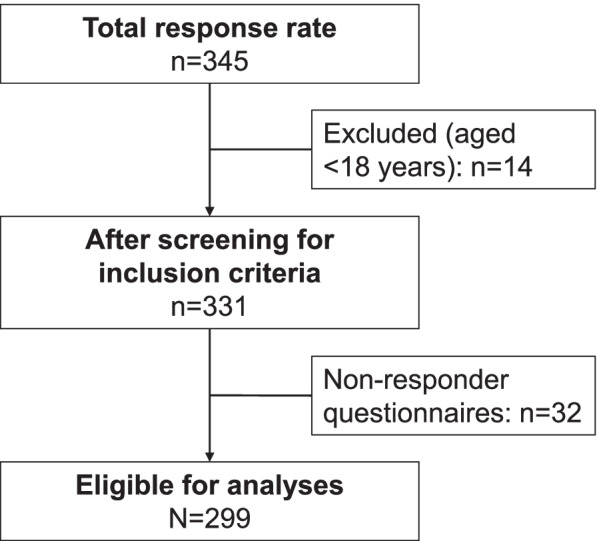
Flow chart of study participants

In the end, ten of the 13 practices that attended the fall research meeting supported the study (practice response: 76.9%). Two practices were not interested in participating, one practice had no time to support the study. The participating practices asked 345 patients to participate (Fig. 1); of these, 32 did not respond (patient response: 90.7%). After excluding participants younger than 18 years who did not meet the inclusion criteria (n = 14), data from 299 primary care patients provided the basis for the analysis.

The participating patients were aged 18 to 88 years (mean: 52.9, standard deviation: 16.3); 173 of them were female (57.9%). More details are provided in Table 1.

**Table 1:** Characteristics of participating patients (*N* = 299)

Female, n (%)	173 (57.9)
Age, mean ± SD (range)	52.9 ± 16.3 (18–88)
Chronically ill, n (%)	133 (45.9)
Employment status, n (%)	
*In training*	13 (4.5)
*Employed*	175 (59.9)
*Seeking work*	14 (4.8)
*Retired*	87 (29.8)
*Other*	3 (1.0)
Reason for practice visit, n (%)^1^	
*Chronic disease*	69 (23.5)
*Acute disease*	95 (32.4)
*Prevention / vaccination*	82 (28.0)
*Other reasons*	91 (31.1)

^1^ *includes multiple responses*

A comparison of the characteristics of responders and non-responders showed that responders were less frequently retired (29.8% versus 50.0%, p = 0.023). They did not differ with regard to age (52.9 ± 16.3 vs. 56.6 ± 20.6 years, p = 0.336), gender (57.9% vs. 53.1% female, p = 0.607), and chronic illness (45.9% vs. 46.4% chronically ill, p = 0.954).

### Relevance of parameters applied in recent studies

**Fig. 2:**
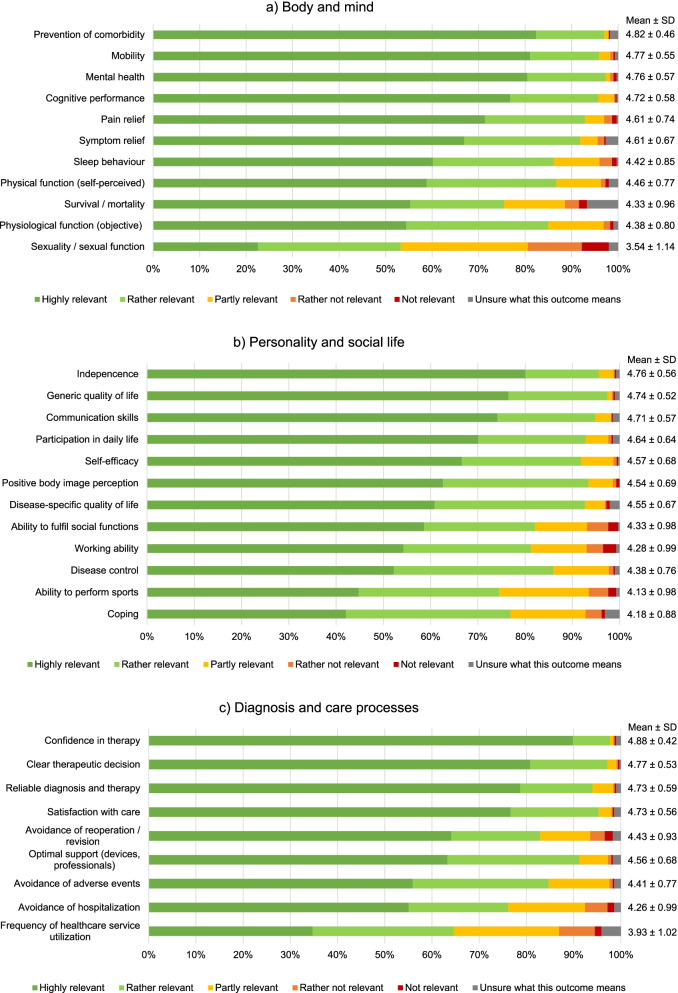
Relevance of supposedly patient-relevant parameters from the patients’ perspective: parameters related to a) body and mind, b) personality and social life, c) diagnosis and care

Within the category *body and mind* (Fig. 2a), more than 75% of all responders rated the parameters ‘prevention of comorbidity’, ‘mobility’, ‘mental health’, and ‘cognitive performance’ as highly relevant. ‘Sexual function/sexuality’ was rated lowest within this category and overall. It was one of only two parameters with an average score lower than 4. In comparison with all parameters assessed, participants were most commonly unsure about what was meant by ‘survival/mortality’ (n = 20, 6.7%).

In the category *personality and social life* (Fig. 2b), three parameters were rated as highly relevant by more than 75% of participants: ‘independence’, ‘generic quality of life’, and ‘communication skills’. In this category all parameters received an average score higher than 4, indicating that all of them were considered relevant.

Within the category *diagnosis and care processes* (Fig. 2c), the four parameters ‘confidence in therapy’, ‘clear therapeutic decision’, ‘reliable diagnosis and therapy’, and ‘satisfaction with care’ were rated as highly relevant by more than 75% of responders. ‘Confidence in therapy’ was also the highest rated parameter overall, considered highly relevant by nearly 90% of participants, and scored 4.9 on average. ‘Frequency of healthcare utilization’ was rated lowest within this category and second lowest overall.

In total, all parameters except ‘sexuality’ and ‘frequency of healthcare utilization’ received an average score higher than 4 and were considered relevant. All mean values and corresponding standard deviations (SD) are presented in Fig. 2a-c.

### Correlations between high relevance and patients’ characteristics

After correcting for multiple testing, comparisons of patients rating a parameter highly relevant with those rating it not highly relevant revealed age- and gender-related differences for five of the 32 parameters, whereas no differences were found for chronic status:

*Age-related differences:* Patients who rated the parameter working ability as highly relevant were significantly younger compared to those not rating it highly relevant (48.9 ± 1.3 vs. 56.8 ± 1.4 years, p < 0.001).

*Gender-related differences:* The proportion of women was significantly higher among those rating cognitive performance (63.2% vs. 39.1% female, p < 0.001), mental health (63.3% vs. 34.5% female, p < 0.001), ability to fulfil social roles (70.0% vs. 39.2% female, p < 0.001), and pain relief (63.2% vs. 42.9% female, p = 0.001) highly relevant when compared with those rating these parameters not highly relevant.

Differences found referred to parameters of the categories *body and mind* as well as *personality and social life*. There were no correlations between high ratings of any parameter addressing *diagnosis and care processes* and patients’ characteristics.

### Redundancies between parameters

The Kaiser–Meyer–Olkin measure of sampling adequacy and Bartlett’s sphericity test indicated sufficient correlations between the variables selected for each PCA. Kaiser’s criteria and scree-plots empirically justified to retain two factors with eigenvalues ≥ 1 for each PCA. The varimax-rotated two-factor solution yielded the most interpretable solution with most parameters loading highly on one of the two factors only (Table 2).

**Table 2:** Principal component analysis

	**Parameters highly loading on only one factor**		**Total variance explained**
Two-factor analysis with parameters related to *body and mind*	Factor 1: -mobility -mental health -cognitive performance -sleep behavior -self-perceived physical function -physiological function	Factor 2: -symptom relief -pain relief	46.4%
Two-factor analysis with parameters related to *personality and social life*	Factor 1: -self-efficacy -positive body image perception -disease control -disease-specific quality of life -coping	Factor 2: -working ability -ability to perform sports -ability to fulfill social roles	50.6%
Two-factor analysis with Parameters related to *diagnosis and care processes*	Factor 1: -clear therapeutic decision -confidence in therapy -satisfaction with care -optimal support -reliable diagnosis and therapy	Factor 2: -avoidance of hospitalization -avoidance of reoperation / revision	60.7%

### Relevant parameters additionally addressed by patients

Thirty-eight of the 299 patients (13.4%) stated that they were missing some relevant parameters in the questionnaire. Of these, one patient did not provide a free-text answer and six answers did not address any specific parameters. The remaining 31 evaluable answers resulted in 15 additional parameters, most of them addressing processes of care and related to the category *diagnosis and care processes*. Those most commonly mentioned parameters were ‘confidence in practitioner’, ‘inclusion of alternative medicine/treatment methods’, ‘enough time in physician consultation’, and ‘being heard’. All parameters added by patients are listed in Table 3.

**Table 3:** Patient-relevant parameters newly addressed by patients (*n* = 31)

Category	Outcome	n (%)
Body and mind	Harmony of body, mind and soul	2 (6.5)
Personality and social life	Empowerment	2 (6.5)
	Financial security (in case of invalidity)	1 (3.2)
Diagnosis and care processes	Confidence in practitioner	9 (29.0)
	Inclusion of alternative medicine / treatment methods	7 (22.6)
	(Enough) time in physician consultation	6 (19.4)
	Being heard	5 (16.1)
	Empathy of practitioner	4 (12.9)
	Being taken seriously	3 (9.7)
	Being understood	2 (6.5)
	Sympathy towards practitioner	2 (6.5)
	Holistic view on patients’ life circumstances	2 (6.5)
	Affordability of health care	2 (6.5)
	Equal health care (independent of income, health insurance)	2 (6.5)
	Time to follow-up treatment	1 (3.2)

## Discussion

The evaluation of parameters identified as patient-relevant in our previous scoping review [6] showed that both parameters addressing processes of care (e.g., ‘confidence in therapy’) and those addressing outcomes of care (e.g., ‘prevention of secondary diseases’) are extremely important to patients. Fifteen parameters were newly identified as relevant. Interestingly, most of them addressed processes of care. Participants assigning high relevance to the parameters cognition, mental health, ability to fulfil social roles, and pain relief were more commonly women, those who consider working ability as highly relevant were younger. No correlations between high ratings of parameters and chronic status were found when correcting for multiple testing. In addition, high ratings of parameters addressing diagnosis and care processes were not associated with any patient characteristics. However, factor analyses revealed that there might be redundancies between some parameters assessed or overlaps between the constructs underlying these parameters.

When comparing the results with other studies that applied patient-driven approaches to define relevant parameters we found that –contrary to our results– parameters addressing processes of care play a secondary role [7–20]. When looking at studies that report patient-relevant parameters for generic patient groups, it appears as though process indicators also play a subordinate role [4, 5, 21–29]. Only few studies have considered such parameters [4, 7, 8, 12, 13, 20, 22, 29]. Those predominantly mentioned are ‘clear therapeutic decision’, ‘confidence in therapy’, ‘reliable diagnosis and therapy’, and ‘optimal support’ [4, 7, 8, 12, 22, 29]. Interestingly, these parameters were mentioned in studies focusing on generic groups as well as in studies focusing on specific diseases. This emphasizes the relevance of these aspects irrespective of the disease, as also indicated by the high scores found in our generic patient sample. Our hypothesis that there are several parameters that are relevant for patients regardless of disease is thus supported.

However, our survey provides only a mere indication of how patients rate the relevance of single parameters without balancing them against each other or providing insight into why some parameters are more relevant than others. It therefore remains unclear how patients ultimately make a choice and which factors or experiences influence their decision or cause their priorities to shift. As our overarching objective is to contribute to an empirically based methodological concept of patient relevance, including a generic set of patient-relevant parameters, it is important for us to understand the processes underlying patients’ decision making. For this purpose, microeconomic approaches might be helpful and need to be considered when developing the concept. Discrete choice experiments or conjoint analyses, for example, are designed to document decisions and are based on the assumption that a higher priority of one option against another also implies a stronger benefit [40]. Implementing such approaches while developing the concept will help us understand what really matters to patients and thereby enable us to derive a framework on how to adequately select and weigh process and outcome parameters when designing a clinical trial and use study results for the process of shared clinical decision-making between doctors and patients. This will be a useful supplement to the planned generic set of patient-relevant parameters.

In order to contribute to the overarching aim of our project, potentially lacking parameters, the relevance of newly identified parameters, the relevance rankings resulting from this survey, and potential redundancies between parameters emerging from the factor analysis will in the next step be discussed with patients, medical and therapeutic professionals, and researchers. Those results will then provide the basis for an online Delphi. The multi-professional approach will ensure that the future concept on patient relevance adequately addresses the patient’s perspective and is feasible for scientific purposes at the same time [31].

### Limitations

One strength of our survey is the high response rate. Nevertheless, we cannot exclude that the results might be limited due to a potential selection bias. Even though we asked the practices to complete non-responder questionnaires for each patient who refused to participate, the number of non-responders is very low, which suggests that the practices might not have consistently completed these questionnaires. Additionally, non-responders differed slightly from responders regarding retirement. Therefore, we cannot finally exclude a selection bias due to systematic study non-response.

One key limitation is that the study sample is small and not representative. In detail, in Germany and in all other European countries the population is slightly younger than the study population and commonly has an almost balanced gender ratio [41], whereas the proportion of chronically ill people is comparable to those reported for Germany and some other European countries [42]. In addition, the parameters assessed were based on a previous scoping review which aimed to identify parameters considered relevant to patients in recent research projects. It cannot be excluded that the review and its search strategy might not have covered all parameters and that the parameters extracted from the studies are incomplete. Due to these limitations the results of this survey only give an indication and provide first insights into how patients rate the relevance of parameters without being generalizable.

## Conclusion

In due consideration of the limitations outlined above, the fact that no correlations were found between chronic status and relevance ratings appears to support the hypothesis that some parameters are relevant to patients regardless of their ailment. In addition, the results of this survey indicate that patients attach great value to parameters that address the process of care. It appears as though the way in which patients experience the care process is not altogether less relevant than its outcome. This is reinforced by the fact that high ratings of parameters addressing processes were not associated with any patient characteristics. However, outcome parameters that have commonly been used in recent studies are still strongly characterized by clinical trial endpoints that focus more on effects of care than on processes of care. This might not adequately represent patients’ reality and satisfy their need for information to make informed choices in the sense of shared decision-making. Addressing patient preferences and parameters that evaluate processes of care from patients’ perspective should become part of the professional self-conception of all researchers and be sufficiently considered when designing studies. In order to harmonize such approaches, there is a need to develop methodological frameworks on how to select and weigh process- and outcome-related parameters when designing trials and that is applicable and adaptable to different contexts.

## Data Availability

The datasets analyzed during the current study are available from the corresponding author on reasonable request.
